# Ultrasound-guided Low Thoracic Erector Spinae Plane Block for Effective Postoperative Analgesia after Lumbar Surgery: Report of Five Cases

**DOI:** 10.7759/cureus.3603

**Published:** 2018-11-16

**Authors:** Sevim Cesur, Ahmet Murat Yayik, Figen Ozturk, Ali Ahiskalioglu

**Affiliations:** 1 Anesthesiology, Regional Training Research Hospital, Erzurum, TUR; 2 Anesthesiology, Ataturk University School of Medicine, Erzurum, TUR

**Keywords:** erector spinae plane block, spinal surgery, postoperative analgesia

## Abstract

Pain control is an important administration of postoperative management in lumbar spinal surgery, and multimodal analgesia is most likely an important strategy in reducing postoperative spinal surgery. Erector spinae plane (ESP) block is a recently described regional anaesthesia technique that blocks the dorsal and ventral rami of the spinal nerves and the sympathetic nerve fibers. While the ESP block has been shown to provide effective postoperative analgesia after thoracic, breast, and abdominal surgery in case reports and randomised controlled studies, there are only a few case series that report that an ultrasonography (US)-guided bilateral ESP block provides effective postoperative analgesia in lumbar surgery. We report five patients undergoing lumbar surgery in which a bilateral lower thoracic ESP block was used as the postoperative analgesia. The bilateral ESP block may be a promising anesthetic method for postoperative analgesia following lumbar surgery. Our aim is testing the safety and efficacy of this technique in various surgical procedures by conducting prospective studies.

## Introduction

Interfascial plane block has recently become very popular for postoperative analgesia management for clinicians. Papers on the erector spinae plane (ESP) block, which was introduced by Forero et al. as a novel fascial plane block in 2016, are on the rise in the literature [1]. ESP appears to be a promising regional method for postoperative analgesia management in thoracic and abdominal surgeries in both adult and pediatric patients [2-4].

Despite the wide spectrum of indications of the technique, there are a few publications in the literature for the use of this technique in lumbar spinal surgery [5].

Our aim in these case series was to investigate bilateral lower thoracic ESP block for providing successful postoperative pain management following lumbar surgery.

## Case presentation

All of the data used in this manuscript were collected after the approval and written consent of all the patients.

**Figure 1:**
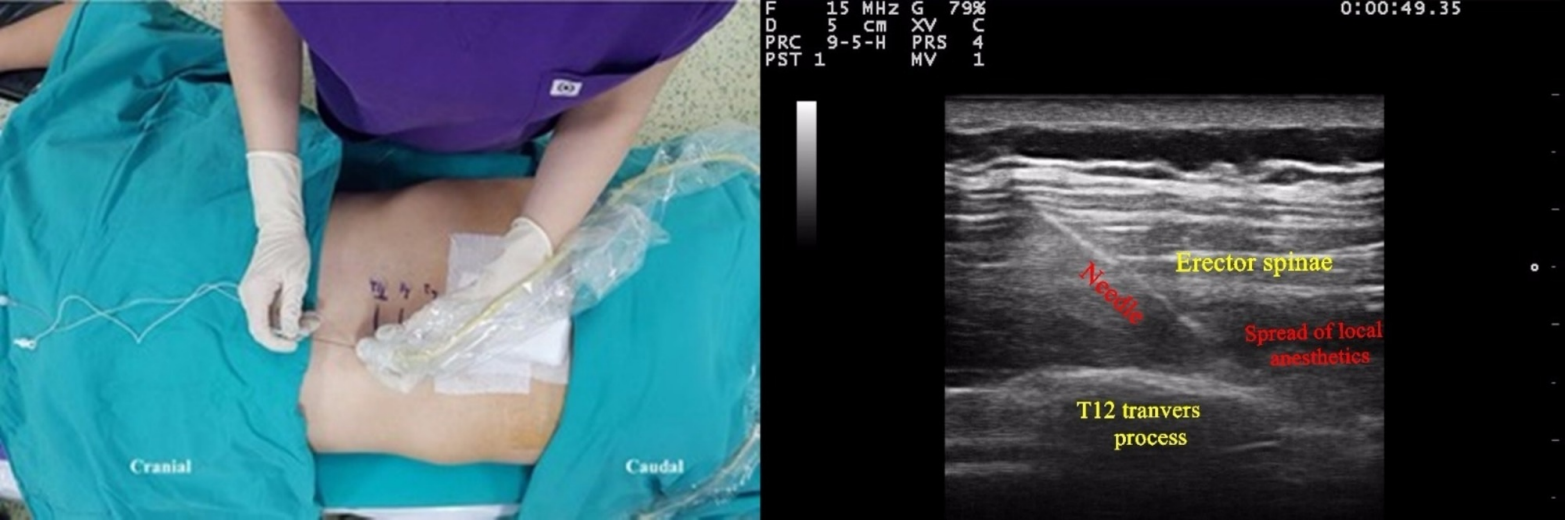
Patient position and sonographic anatomy of ESP ESP: erector spinae plane

All patients were positioned in a prone position for preoperative ESP block application. The skin was prepared with chlorhexidine 2% in a sterile fashion. The ESP block was performed using an Esaote MyLab™ 30 US machine (Esaote SpA, Florence, Italy) with a large bandwidth and multifrequency linear probe (10–18 MHz) of 22G-80 mm containing an insulated facet type needle (Braun Sonoplex, Melsungen, Germany). The ultrasonography (US) probe was placed on the spinal column at the level of T12 laterally by means of the guidance of the twelfth rib (Figure 1). The erector spinae muscle and the transverse process were seen. After negative aspiration, the needle position was confirmed by the administration of a normal saline solution. A 20 ml local anesthetic solution containing bupivacaine 0.25% and lidocaine 1% was injected deep into the erector spinae muscle in the course of the direction from cranial to caudal.

Each patient was taken to the operating room after the dermatomal spread.

All patients received 400 mg intravenous (IV) ibuprofen in a 100 ml saline solution 30 minutes before surgery. For all the patients, a standard monitorization method was applied. Anaesthesia was induced with propofol 2 mg/kg, fentanyl 2 mcg/kg, and rocuronium 0.6 mg/kg. Each patient was then intubated with a spiral cuffed endotracheal tube (ETT) (women with 7.5 no ETT; men with 8.0 no ETT). Anesthesia maintenance was achieved with a desflurane 6% combination with nitrous oxide in oxygen at a proportion of 2:1 in 3 liters (L) of fresh gas flow. The patients were ventilated to maintain an end-tidal carbon dioxide level of 35 - 40 mmHg. Radial artery catheterization was performed in the non-dominant hand of all patients using a 20 G catheter for invasive blood pressure monitorization and arterial blood gas (ABG) analysis after induction. The hemodynamics of each patient were closely followed during surgery with no complications. At the end of the surgery, tramadol-based patient-controlled analgesia (PCA) was administered before extubation. The patients were then extubated and taken to the postoperative recovery unit. The Numeric Pain Rating Scale (NPRS) for pain was recorded every hour during the 24-hour postoperative follow-up period. Please refer to Table 1 below for a summary of clinical details for all five patients.

**Table 1:** Summary of the Five Patients in This Case Series NPRS: Numeric Pain Rating Scale ranges from 0 (no pain) to 10 (worst pain imaginable); ASA 1 and 2: American Society of Anesthesiologists Scores

	Case 1	Case 2	Case 3	Case 4	Case 5
Demographic/ clinical details	52-year-old male, 80 kg, 172 cm, ASA 1	60-year-old female, 76 kg, 155 cm, diabetes mellitus, ASA 2	48-year-old male, 88 kg, 176 cm, hypertension and diabetes mellitus, ASA 2	34-year-old female, 67 kg, 156 cm, ASA 1	57-year-old male, 84 kg, 180 cm, significant smoking history
Surgery type	Lumbar disc herniation (L3-4)	Lumbar instrumentation (L2-4)	Lumbar disc herniation (L2)	Lumbar disc herniation (L4)	Lumbar instrumentation (L3-5)
Duration of surgery	2 hours	4 hours	2 hours	1 hour	3 hours
NPRS postop 4 hours	0	3	2	0	3
NPRS postop 8 hours	2	4	2	0	3
NPRS postop 12 hours	2	4	2	2	4
NPRS postop 24 hours	4	5	3	2	4
Postoperative total tramadol consumption use	60 mg	150 mg	45 mg	30 mg	100 mg

## Discussion

Pain control is an important direction of postoperative management in lumbar spinal surgery. Inadequate pain control increases cardiac and respiratory complications, as well as delays mobilization, increasing the length of hospital stay and the risk of chronic pain syndrome [6]. For all these reasons, multimodal analgesia is most likely an important strategy in reducing postoperative pain following spinal surgery [7]. Opioids are known to play a role in moderate-to-severe pain management, while NSAIDs are effective in pain management where inflammation is the cause. However, the side effects of opioids, such as respiratory depression, nausea, vomiting, and pruritus, should also be considered. Zhang et al. reported that the use of NSAIDs in lumbar spinal surgery was effective in postoperative pain management in a meta-analysis, and the surgery type and NSAIDs dosage have an important role in the analgesic effect. Systemic methods, as well as regional methods, are an important step in multimodal analgesia. Neuraxial blockade includes epidural and spinal blocks managed via a single bolus, continuous infusion, or patient-controlled delivery systems. Although neuraxial blockade reduces opioid consumption and side effects, these blocks may cause hemodynamic instability and motor block [8]. Recently, interfascial plan blocks have started to be involved in postoperative pain management after lumbar surgery [9-10]. Interfascial plane blocks reduce opioid consumption without motor blocks, such as neuraxial blocks, providing adequate long-lasting postoperative analgesia [11]. 

The newest interfascial plane block, the ESP block, is a block made by injecting local anesthesia between the deep fascia of the erector spinae muscle and the transverse process and targeting the dorsal and ventral rami of the spinal nerves. Although studies have not completely defined the mechanism of the ESP block, the analgesic efficacy of the ESP block is thought to be due to local anesthetics spreading to the paravertebral space [1, 12].  

The ESP block has provoked the curiosity and interest of clinicians as the paravertebral distribution associated with the block has been established and the method is relatively easier and safer to apply compared to the paravertebral block. Thus, the publication of data on the successful management of postoperative analgesia by the application of the ESP technique at the level of T5 after the thoracic and breast surgeries has been followed currently by reports on the use of that method at various levels for a range of indications from acute herpes zoster treatment at low thoracic levels to abdominal and hip surgeries [13-15].

In this case series, we observed that the ESB achieved effective analgesia and reduced opioid consumption in the single or multilevel lumbar spine surgeries of five patients during the postoperative first 24 hours.

## Conclusions

In conclusion, on the basis of this small case series, it appears that bilateral US-guided ESP block is a safe and effective technique for postoperative pain management after lumbar spine surgery.

We recommend testing of the safety and efficacy of this technique in various surgical procedures by conducting prospective studies.
